# Advances of Nano-Structured Extended-Release Local Anesthetics

**DOI:** 10.1186/s11671-019-3241-2

**Published:** 2020-01-16

**Authors:** Yumiao He, Linan Qin, Yuguang Huang, Chao Ma

**Affiliations:** 1 Department of Anesthesiology, Peking Union Medical College Hospital, Chinese Academy of Medical Sciences, Beijing, 100730 China; 2 Joint Laboratory of Anesthesia and Pain, Peking Union Medical College, Beijing, 100730 China; 3 Department of Human Anatomy, Histology and Embryology, Institute of Basic Medical Sciences, Chinese Academy of Medical Sciences, School of Basic Medicine, Peking Union Medical College, Beijing, 100005 China

**Keywords:** Extended-release, Local anesthetics, Nano-scale, Liposomes, Polymersome, Hybrid

## Abstract

Extended-release local anesthetics (LAs) have drawn increasing attention with their promising role in improving analgesia and reducing adverse events of LAs. Nano-structured carriers such as liposomes and polymersomes optimally meet the demands of/for extended-release, and have been utilized in drug delivery over decades and showed satisfactory results with extended-release. Based on mature technology of liposomes, EXPAREL, the first approved liposomal LA loaded with bupivacaine, has seen its success in an extended-release form. At the same time, polymersomes has advances over liposomes with complementary profiles, which inspires the emergence of hybrid carriers. This article summarized the recent research successes on nano-structured extended-release LAs, of which liposomal and polymeric are mainstream systems. Furthermore, with continual optimization, drug delivery systems carry properties beyond simple transportation, such as specificity and responsiveness. In the near future, we may achieve targeted delivery and controlled-release properties to satisfy various analgesic requirements.

## Introduction

Pain has been regarded as “the fifth vital sign” since 1996 due to its significance in physical and mental health [1]. In order to deal with opioid crisis happening during traditional pain management, concept of multimodal analgesia is introduced. Local anesthetics (LAs) is one of the most frequently-used and safest analgesics in multimodal regimens [2, 3]. However, limited duration (less than 24 h) and potential toxicity (cardiac and central nervous malfunction) restrict its application and raise the urgency to counterbalance the side effects and prolonged analgesia [4–6]. Although disposable catheters with pumps are used to prolong LAs’ duration, the risks of catheter dislodgement, infection, and trauma during procedure do exist. Additionally, catheter placement is labor- and time-consuming [5]. Extended-release LAs compensate for the aforementioned disadvantages. They are capable of continuously releasing a safe dose with single administration (usually injection without general anesthesia, requiring minimal invasive techniques and special tools) to assure minimal systemic toxicity. Meanwhile, prolonged duration of nociceptive block can be achieved.

Compared to macro-scaled drug delivery systems (DDSs), nano-scaled DDS is more compatible to nano-structured biological environment which facilitates its cellular penetration, better bioavailability, and longer retention time [4]. With advances of manufacture techniques, nano-scaled DDS have achieved prominent success in extended-release, which show improved loading efficiency, better biocompatibility (acceptable local inflammation such as myotoxicity and neurotoxicity), and biodegradability (similar degradation rate with depletion of loaded compounds and assuring fully wearing off) [7–9]. Furthermore, adjustable and differentiated release profile (flexible duration, modulated analgesic intensity, and controlled targeted release by specific modifications) is designed to meet different demands [7, 8]. Furthermore, diversity of materials are available for choice nowadays, greatly decreasing the cost and expanding the application of nano-scale DDS [7].

However, nano-scaled DDS still faces drawbacks, such as burst release due to high surface-volume ratio, poor stability of liposomes on shelf, and un-ineligible foreign body response of synthetic polymers [4], all of which attract increasing efforts to optimize nano-scaled DDS further. Here, we summarize several state-of-the art formulations of nano-structured extended-release LAs. The most commonly used morphology is nanoparticle, while nano-structured gel, niosome, film, and rod are accessible as well [8, 10, 11]. Here, synthesis techniques, release profiles, analgesic effects, and safety quality are provided and compared to help future design and development.

## How Do Local Anesthetics Work?

### Analgesic Mechanism and Physiochemical Properties

**Fig. 1:**
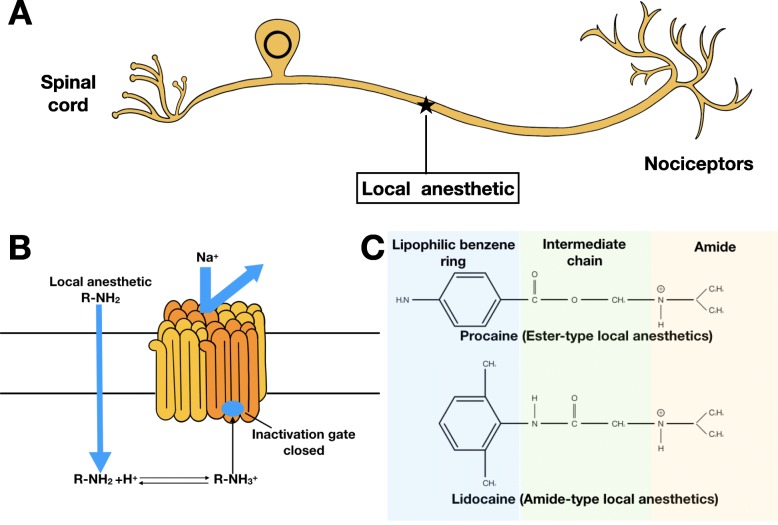
Functional and Structural Properties of LAs. **a**, **b** Demonstration of how LAs interact with voltage-gated sodium channel on neuron. **c** Typical structures of ester and amide LAs

Peripheral nerves are the first stops to perceive pain stimuli during pain transmission [12]. It is reasonable to consider inhibiting pain from the very beginning, stopping downstream reactions and maladaptive changes of neuroplasticity which are more difficult to control [13]. Therefore, LAs become a perfect choice. LAs work on peripheral nerves via binding to intracellular domain of voltage-gated Na channel, inhibiting influx of Na^+^, resulting in the blockade of depolarization [14] (Fig. 1a, b).

LAs are constituted with three chemical groups: a hydrophilic amino group (mostly tertiary amines), a lipophilic benzene ring, and a linker which can be an amide or an ester, determining LAs’ classification (Fig. 1c). Amide-type LAs are the most commonly used, including bupivacaine, ropivacaine, lidocaine, and mepivacaine. Ester-type LAs involve chloroprocaine, procaine, and tetracaine [14, 15]. Pharmacokinetics such as speed of onset, potency, and duration are largely determined by its physiochemical properties. Permeability through neuronal membrane is a decisive factor for onset which is influenced by the equilibrium between charged and uncharged LAs. With pKa closer to extracellular pH, greater amount of uncharged LAs are formed and diffused into neurons to take effect. Potency of LAs, also called analgesic efficacy, is a result of lipophilicity, which can be quantified by the partition coefficient. Duration is a representative of protein binding affinity, which also creates a reservoir as free LAs are metabolized. Overall, ester-type LAs show relatively rapid onset due to its physiologically similar pK_a_, but short duration because of easier hydrolysis and poorer protein affinity in vivo, while bupivacaine is the LA with the longest duration of effect due to its long alky chain [14, 16] (Table 1).

**Table 1:** Characteristics of LAs

Local anesthetic	Class/chemical linkage	Onset time	Potency	Duration of action/min
Lidocaine	Amide	Fast	Moderate	120 [14]
Bupivacaine	Amide	Slow	Potent	240 [14]
Ropivacaine	Amide	Slow	Potent	240 [17]
Prilocaine	Amide	Fast	Weak	120 [14]
Mepivacaine	Amide	Fast	Moderate	120 [14]
Articaine	Ester	Fast	Moderate	60 [18]
2-Chloroprocaine	Ester	Very Fast	Moderate	60 [14]
Tetracaine	Ester	Fast	Moderate	120 [14]
Procaine	Ester	Slow	Weak	30 [14]

### Systemic Toxicity

Properties determining effects are also related with systemic toxicity. In spite of blood-brain-barrier (BBB), LAs enter central nervous system (CNS) readily with proper molecular weight, pKa, and good lipophilicity. LAs with low molecular weight, high lipid-solubility, and appropriate pKa such as lidocaine and procaine show rapid parallel change of concentration in spinal-cerebral liquid to plasma drug concentration [19]. Because LA-Na_v_ channel binding is non-exclusive, when LAs are leaked into cardiovascular system accidentally, organs with profuse blood perfusion and high activity of voltage-gated channel (Na, K, Ca), such as heart and brain, will be attacked preferably by LAs leading to organ malfunction [20, 21]. Besides the inhibition to excitation conduction of vulnerable organs, energy depletion due to mitochondrial dysfunction and apoptosis contribute as well [15, 22].

Therefore, the overall toxicity is resulted from extracellular drug concentration, cell membrane permeability, and toxic reactions induced, which can explain the more severe CNS toxicity of quaternary derivative QX-314 compared to lidocaine, although QX-314 penetrates BBB and cell membrane slower [23]. In contrast to central nervous presentations (convulsion and seizure) which are more common, but relatively easier to control, cardiac malfunction, such as conducting disturbance, arrhythmia, and contractile dysfunction, can cause deadly outcome [6, 15]. In order to prolong LAs’ analgesic effect while preventing adverse events, many efforts have been put into the development of extended-release LAs.

## Liposomal Formulation of Extended-Release LAs

### General Ideas about Liposome

**Fig. 2:**
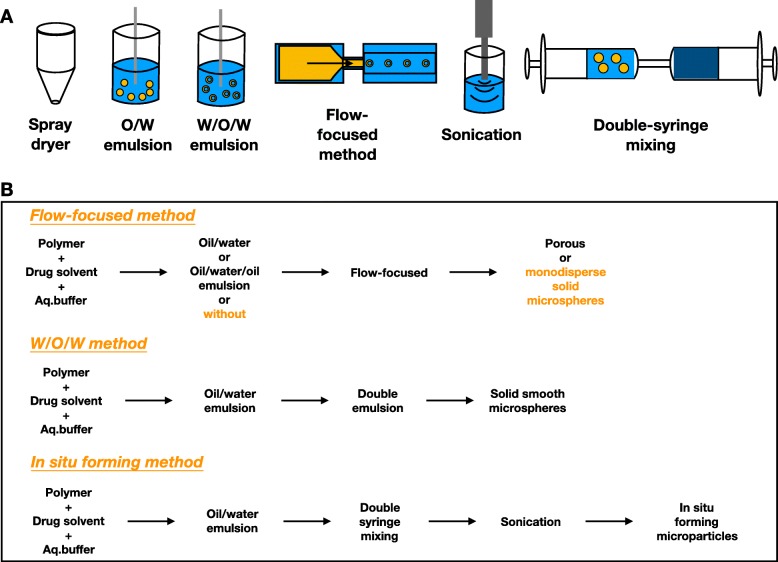
Classical techniques of producing liposomes. **a** Basic production techniques for liposomes. **b** Demonstrations of production workflow

**Fig. 3:**
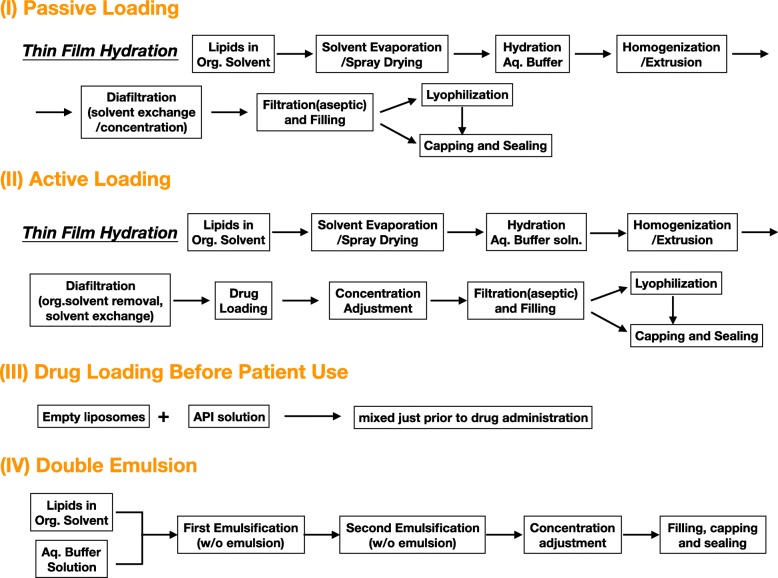
Various drug-loading pathways

**Fig. 4:**
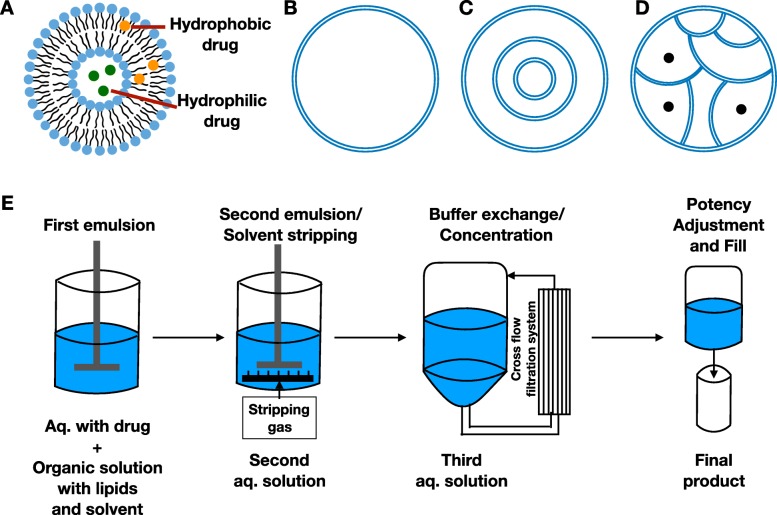
Structural characteristics of different liposomes. **a** Drug distribution in liposomes. **b** Uni-lamellar liposome. **c** Multi-lamellar liposome. **d** Multi-vesicular liposome. **e** Demonstration of DepoFoam technique

Liposome is a lipid vesicle of nanometer scale, which is phospholipid-based [10]. Liposomal techniques have been long utilized in drug delivery for treating diseases such as cancer, infection, and eye disease. Lipid has a hydrophilic head and hydrophobic tail which are linked by ester or ether bond [24]. Liposomes are produced by aggregation of lipid units in a bilayer form, creating an aqueous sheath and core with hydrophilic heads, shielding hydrophobic tails inside layers. From simple sonication to more sophistic dry-spying technique, increasing innovative approaches have been developed based on diverse combinations of sonication, emulsion, dry-spraying, and flow-focusing to produce liposomes with improved properties(Fig. 2) [24–28]. Thin film hydration is one of the most commonly used production techniques, while microfluidic technique is promising to scale up [29]. Drugs can be entrapped through different ways including passive loading, active loading, loading before patient use, and double emulsion (Fig. 3). Finally, drugs are carried in the aqueous core or between lipid layers depending on the drugs’ hydrophilicity (Fig. 4a). This generous compatibility enables liposomes competent in delivering various drugs [24].

Several factors should be considered when building a stable and effective controlled-release system. Lipid composition is the basic and pivotal factor for designing liposomes. First of all, a stable suspension system should be assured which means no aggregation and fusion occur. Composition is the first attribute. Surface charge and electrostatic force can help liposomes repulse against each other to avoid aggregation [30]. However, suspension environment can neutralize surface charge to induce aggregation. 1,2-Dipalmitoyl-sn-glycero-3-phosphate (DPPA) and 1,2- distearoyl-sn-glycero-3-phospho-(1′-rac-glycerol) (DSPG) are two negatively charged lipids belonging to phosphatidylserines, which tend to aggregate when encountering calcium and magnesium cations [31]. Therefore, liposomes containing higher proportion of negatively charged lipids have a higher tendency to aggregate upon addition of divalent cations, which can help determine the lipids’ proportion and cation concentration in liposomal suspension. With determined components, particle size is another attribute to aggregation. Larger particles are more likely to aggregate in spite of net charge [30]. Besides ionic components, another environmental factor is temperature. Low temperature will put liposomes in closer proximity, inducing aggregation. Temperature slightly above transition temperature is recommended for storage [30, 32]. Another strategy to avoiding aggregation is to protect liposomes from interacting with each other by being coated with polyethyleneglycol (PEG). This hydrophilic molecule can work as a sheath, not only to prevent aggregation but also to broaden the choices of surface modification [33]. Building on this idea, improvements have been made to optimize the protection such as the addition of insulin and dextran, and PEGylated bolaamphiphile [34, 35]. Fusion needs more attraction force which is less common to happen compared to aggregation because hydration repulsion force exists when electrostatic force is absent [31]. And this form of repulsive force can be strengthened by increasing proportion of phosphatidylcholine [30, 31].

As for controlled release, decreased ratio of unsaturated lipid tails, more long-chain lipids, and ether linkers can promote stability of liposomes and slow release down. Inserting cholesterol and decorating with molecules such as PEG can also stabilize liposomes which can be classified as hybrid as described later in the review. These modifications decrease fluidity and biodegradation of liposome. Surface charge and particle size are another two considerations. Liposomes of neutral charge and smaller size are cleared more slowly [24, 36]. Octanol/buffer partition coefficient is a characteristic of actives indicating the distribution of drugs when liposomes enter physiological environment, which also determines release pattern. Drugs like bupivacaine with high octanol/buffer partition coefficient can be easily released out [16, 36].

Components not only influence physiochemical properties of liposomes but also influence physiological properties, such as tissues specificity. When liposomes enter blood circulation, phagocytosis by macrophages is the main clearance pathway. However, different recognitions by macrophages in reticuloendothelial systems (RES) happen due to different opsonization process, of which lipid composition, particle size, and surface charge are vital determinants [37, 38]. For example, activation of complement system primarily determined by cholesterol is recognized by Kupffer cells around portal area in liver, while saturated phospholipids enhance recognition by macrophages in spleen and bone marrow [39]. Particle of larger diameter (~ 100 nm)is cleared preferentially by Kupffer cells [37, 38]. Moreover, negatively charged liposome is selectively recognized by scavenger receptor on Kupffer cell while neutral liposomes are more indolent [38]. The phagocytosis occurs rapidly, while the intracellular process is slow ranging from hours to months which results in prolonged retention in RES [37, 38]. This retention can help target RES while hindering systemic distribution. Several strategies have been developed. Morphology of filamicelle can limit the accessibility of binding sites between liposomes and receptors. Soft liposomes are less preferred by macrophages. Stealth strategy by PEGylation and CD47 modification can also passivate liposomes [40]. Saturation of macrophages (RES blockade) by extra liposomes is an alternative to extend circulation of drug-loaded liposomes [41]. In a more aggressive way, transient macrophage depletion can be performed by clodronate.

After escaping from clearance by macrophages, liposomes show good biocompatibility and bio-distribution. For tissues with high vasculature, profound blood perfusion and large vascular pore, liposomes have great tendency to enter. Tumor tissues exhibit all of the properties mentioned above. With aberrant vascular formation and lymphatic drainage, liposomes can easily enter and stay longer in tumor tissues, which is called enhanced permeability and retention (EPR) [42, 43]. Furthermore, liposomes with larger size and positive charge can overcome high interstitial fluid pressure in the center of tumor and penetrate it thoroughly [42]. Apart from passive targeting by blood transportation and structural advantages, active targeting by surface modification is also used widely to optimize selectivity, such as epidermal growth factor receptor (EGFR)-ligand and vascular endothelial growth factor (VEGF)-ligand for tumor targeting, and transferrin ligand and sphingomyelin for BBB penetration [42, 44, 45].

### Multivesicular Liposome

Different packings of lipid vesicles can also help prolong drug release. Usually, liposomes are concentric where lipid membranes are packed in a multi-lamellar or uni-lamellar way (Fig. 4b, c) [16]. Therefore, collapse of internal lipid membrane will lead to drug accumulation of drug and rapid release following breach of external layer [46]. In this circumstance, sustained release cannot be achieved. Multivesicular liposomes are non-concentric where vesicles are closely packed to each other from outside (Fig. 4d). Double emulsion, which is also called DepoFoam technology, is used in the production of multivesicular liposome (Fig. 4e). Regarding multivesicular structure, at least one neutral lipid (triglyceride) and one amphipathic lipid (phospholipid) should be chosen. The percentage of triglyceride is related with drug loading efficiency within a certain range [46, 47]. It achieves longer release through gradual degradation of outermost vesicles. Multivesicular structure remains during rearrangement of internal vesicles which is isolated by outer vesicles from external environment to avoid burst release [46, 48]. Multivesicular liposomes show great dominance in release duration, which can release drug over several days to weeks through non-vascular administration compared to hours to days by uni-lamellar and multi-lamellar liposomes through intravascular route [46, 49].

### Additional Advantage

Compared to other encapsulating carriers, liposomes may possess additional advantages when delivering LAs. As known by all, lipid emulsion therapy has shown its efficacy in dealing with systemic toxicity of LAs and been added into resuscitation guideline of LAs’ toxicity [50]. The potential mechanisms include releasing LAs from Na_v_ channels in myocardial cells, partitioning and redistributing LAs to storage (fatty acid), detoxification (liver) and excretion organs (kidney), providing extra energy, and augmenting Ca channels to strengthen cardiac output [51, 52]. Although liposomes are assumed to release bupivacaine through erosion and degradation, simultaneous diffusion through lipid membrane happen which can maintain the integrity of liposomes [16, 53, 54]. There is a research finding that large fraction of liposomes can retain its multivesicular structure even after drug is released near-completely in vitro indicating diffusion may dominate [46, 55]. Furthermore, ultrasound-induced pore formation can help remain size and structure of liposomes unchanged [56]. Therefore, assumption can be made that bupivacaine possibly shows decreased toxicity in the form of liposomes with the protection of lipids. Further researches and design manufacturing are needed to confirm and maximize this benefit.

Liposomes have been used in brain-directed drug delivery due to good biocompatibility and lipophilicity [57, 58]. There are several mechanisms mediating penetration through BBB. Transcytosis mediated by receptor or absorption is the main pathway for crossing BBB [45]. Absorption is largely determined by electrostatic attraction between liposomes and negatively charged cell membrane [45]. When liposomes are small enough, such as unilamellar liposomes encompassing lipophilic drugs, passive diffusion may occur. Modifications are also designed to strengthen ligand-receptor binding to facilitate brain-directed drug delivery [57]. Therefore, reverse designs such as negative net charge and lack of BBB-specific ligand can help prevent brain penetration and enhance safety.

### EXPAREL-First Liposomal LA Approved

EXPAREL (Pacira Pharmaceuticals, Parsippany, New Jersey, USA) is the only extended-release liposomal LA which is approved by American Food and Drug Administration (FDA). The liposomes suspension is produced by DepoFoam technology with tricaprylin which belongs to neutral lipids, and amphipathic lipids, including 1,2-dipalmitoyl-sn-glycero-3-phospho-rac-(1-glycerol), 1,2-dierucoylphosphatidylcholine, and cholesterol [47, 49]. It is allowed legally in limited clinical situations, including wound infiltration which was approved in 2011, and interscalene brachial plexus block in 2018. It can achieve prolonged release and analgesic efficacy up to 72 h [5].

Compared to conventional analgesic regimen, such as free LA injection, pain pump, epidural analgesia, and patient controlled analgesia (PCA), EAPAREL has demonstrated its non-inferior analgesic potency when used through wound infiltration. Besides on-label indications, EXPAREL has also shown promising role in other analgesic routes, such as intercostal nerve block, transversus abdominis plane (TAP) block, and satellite ganglion block (Table 2). However, there are researches showing negative results on analgesic potency of EXPAREL regarding wound infiltration. These researches are different in surgeries of distinct pain intensity, also in the time-points of pain evaluation, which are relatively earlier such as 12 h. Orthopedic surgeries usually lead to severe pain in patient, therefore single wound infiltration with EXPAREL may be insufficient in controlling pain. Compared to plain bupivacaine, EXPAREL may not release adequate bupivacaine at the initial time and cannot be co-administrated with free LAs additionally to achieve better analgesia (Table 3). Therefore, analgesic effect of EXPAREL may depend on the type of surgery, pain intensity, and expected analgesic onset.

**Table 2:** Summary of researches with positive efficacy of EXPAREL

Reference number	Route	Comparison	Surgery	Primary outcome	Results
[59]	Injection into the trocar path and vaginal incision	Saline	Retropubic midurethral sling	VAS pain score 4 hours after discharge home	Pain score was lower in intervention group (*n* = 54, 3.5), than in control group (*n* = 55, 3.5) (*p* = 0.014)
[60]	Interscalene block	Continuous interscalene nerve block with plain bupivacaine	Shoulder arthroplasty	Pain assessment up to 24h after surgery, all doses and times of narcotics during the inpatient stay	No significant difference for primary end point; LB group (*n* = 34) had higher American Shoulder and Elbow Surgeons score (74.5) and Penn Shoulder Score (72.3) than control (*n* = 32, 59.7, 56.3) at final follow-up
[61]	Posterior intercostal nerve block	Thoracic EPI	Lung resection	Perioperative morbidity, pain scores and narcotic requirements	Non-inferior analgesia of LB group compared to control (*n* = 54 respectively)
[62]	Multilevel intercostal nerve block	EPI	Open thoracotomy	Mean pain score on POD 1, 2, 3, supplemental narcotic utilization, total length of hospital stays	LB group (*n* = 53) showed lower mean pain score on day 1 (*p* < 0.04) and 3 (*p* < 0.04) compared to EPI (*n* = 32), the length of hospital stay was longer in LB group (7.4 days) compared to EPI group (9.3 days) (*p* < 0.05)
[63]	Intraoperative intrathoracic intercostal nerve rib blocks	Thoracic with bupivacaine hydrochloride	Video-assisted thoracoscopic pulmonary resection	Pain score, postoperative opioid medication	LB group had significantly lower VAS scores (*n* = 143, 3.9 versus 4.5, *p* < 0.05), decreased postoperative opioid medication (morphine equivalent dose during the first 3 days: 344.5 versus 269.5, *p* < 0.05) than control (*n* = 237)
[64]	Post-incisional TAP	Plain bupivacaine infiltration	Bariatric Surgery	All narcotics used	LB group (*n* = 233, 44.5mg) required less narcotic than control group for entire hospital stay (*n* = 243, 78.0mg) (*p* = 0.00001)
[65]	TAP	Pain catheter (OnQ)	Delayed unilateral deep inferior epigastric perforator reconstruction	Intravenous, oral and total narcotics utilization	LB group (*n* = 6) compared to OnQ group (*n* = 6) used 19.3 mg vs. 29.6 mg intravenously, 40.9mg vs. 53.2mg in total (*p* = 0.005, < 0.001= respectively
[66]	TAP	Intravenous patient control analgesia (IV PCA), EPI	Major lower abdominal surgery	Total postoperative IV morphine-equivalent dose of opioid and time-weighted average NRS pain scores	TAP infiltration (*n* = 108) was noninferior to EPI (*n* = 108)on both primary outcomes (*p* < 0.001)
[67]	TAP	0.25% bupivacaine injection	Laparoscopic hand-assisted donor nephrectomy	Maximal pain scores, opioid consumption at 24, 48, 72 h postoperatively	LB group (*n* = 30) compared with control (*n* = 29) median, showed lower pain scores on 24–48 h after injection (5 vs. 6, *p* = 0.009); on 48–72 h after injection (3 vs. 5, *p* = 0.02); and fewer opioid use on 48–72 h after injection (105 vs. 182, *p* = 0.03)

*POD* postoperative day, *VAS* visual analog scale, *NRS* numeric rating scale, *LB* liposomal bupivacaine, *EPI* epidural analgesia, *TAP* transversus abdominis plane block

**Table 3:** Summary of researches with negative efficacy of EXPAREL

Reference Number	Route	Comparison	Surgery	Primary Outcome	Results
[68]	Periarticular injection	Standardized cocktail	Total knee arthroplasty	VAS, total-morphine-equivalents (TME), and opioid-related-symptoms-distress-Scale (OR-SDS) at 24 and 48 h postoperatively	The LB group (*n* = 52, TME = 51.5) required significantly more narcotics than control (*n* = 52, TME = 30.03), *p* = 0.025
[69]	Periarticular injection	Perioperative nerve block, ropivacaine periarticular injection	Total knee arthroplasty	Maximal NRS in intention- to-treat analysis	Median maximal pain scores were significantly lower for peripheral nerve blockade (*n* = 50) compared to LB group (*n* = 52) (*p* = 0.016)
[70]	Periarticular injection	Femoral nerve block	Anterior cruciate ligament reconstruction	Postoperative VAS for 4 days	A significant increase in pain in LB group (*n* = 41) between 5 and 8 h compared to control (*n* = 41) postoperatively (6.3 ± 2.0 versus 4.8 ± 2.6; *p* = 0.01)
[71]	Posterior vaginal wall infiltration	Saline	Posterior vaginal wall surgery	Morphine equivalent narcotic usage, pain score on postoperative day 1, 3, 7	No difference observed (study group *n* = 49, control group *n* = 51)
[72]	Parasternal nerve block	Saline	Median sternotomy for coronary revascularization	Total amount of narcotic pain medication used, patient`s pain score within the first 72 h postoperatively	No difference between two groups (study group *n* = 38, control group *n* = 41)
[73]	Intraoperative local infiltration	Preoperative interscalene nerve block	Shoulder arthroplasty	Postoperative average daily VAS scores for 4 days	Significant increase in pain in the LB group (*n* = 26) between 0 and 8 h postoperatively (5.3 vs. 2.5, *p* = .001) compared to control (*n* = 31)
[74]	Infiltration	Interscalene Block	Shoulder arthroplasty	Morphine equivalent units (MEU) consumed over the first 24 h	Similar MEU over the first 24 h; intraoperative narcotics and mean VAS pain score were significantly lower in control than in LB group (8.9 ± 4.1 vs. 16.2 ± 7.0 MEU *p* < 0.001; at 0 h 0.8 ± 2.2 vs. 3.3 ± 2.7 points, *p* < 0.001; at 8 h 1.4 ± 2.4 vs. 3.2 ± 2.2 points; *p* < 0.001). (*n* = 78 in all groups)

*VAS* visual analog scale, *NRS* numeric rating scale, *LB* liposomal bupivacaine

In spite of controversy in analgesia, administration route of EXPAREL is simpler and safer compared to catheter insertion, nerve block, and epidural analgesia [62, 75, 76]. In animal models, EXPAREL does not induce neurotoxicity (reduction of neuronal concentration or demyelination) after peri/intraneural or subarachnoid injection [77–79]. But EXPAREL-induced regional inflammation around injection site is higher compared to conventional bupivacaine HCl, but similar with saline [78]. Overall, EXPAREL shows similar toxicity with free bupivacaine [80–83]. Medical cost using EXPAREL decreases further compared to epidural analgesia [63, 84] and continuous pump [60], but the cost is greater than plain LAs injection [85–88]. Therefore, utilization of EXPAREL needs comprehensive considerations including different requirement of analgesia expected in different surgeries and cost-efficiency.

### Attentions

EXPAREL has an obvious restriction on application due to fluidity and rearrangement of lipid layers. Rapid release of bupivacaine with additional use of free LAs, especially within 20 min, will occur due to replacement of bupivacaine in liposomes [89]. In this circumstance, incidence of local anesthesia systemic toxicity (LAST) will increase dramatically. Lidocaine especially shows stronger affinity to DepoFoam, inducing greater systemic exposure of both lidocaine and bupivacaine when co-administered with EXPAREL within 20 min. However, the risk of burst release is not only due to replacement but also due to vascular effects of LAs and vasoconstrictors used, and diluting/mixing effects. Therefore, time beyond 20 min assures a safe level both of EXPAREL and free LAs [90]. As for bupivacaine HCl, a mixture with EXPAREL is allowed of a ratio lower than 1:2 to assure safe and sustained release [5, 49]. Since analgesia is a multi-disciplinary cooperation, careful attention should be paid to administer EXPAREL. Moreover, all liposomal products have a storage problem which is described as unstable “on the shelf.” Lipids will be degraded into harmful metabolites over a long period of storage. Excessive lysolipids and other lipid debris will bind to red blood cells resulting in deadly hemolysis. Being coated with chitosan or alginate may overcome this problem by stabilizing liposome and extending conservation up to 2 years [24, 36].

### More Inspirations

Efforts from other points are also taken to improve liposomal bupivacaine’s analgesic efficacy. Christopher Weldon et al. increased cell-liposomal bupivacaine interaction through decreasing particular size to ten times smaller, prolonging its regional retention which achieved longer anesthesia and similar safety quality compared to regular liposomal bupivacaine in Bier block [91]. Changyou Zhan and his colleagues combined gold nanorods and liposomes to achieve phototriggered anesthesia. This photo-reactive system minimizes the required single dose of near-infrared light and exhibits satisfactory safety. More importantly, it achieves on-demand and repeated regional anesthesia, which will make pain management more individualized and precise [92]. Alternatively, strengthening interaction of LAs and lipids with evidence that LAs has different affinity with different lipids [93], and encapsulating liposomes with alginate to enhance anti-inflammatory reaction of mesenchymal stromal cells [94], may further improve controlled-release and meet different clinical demands.

## Polymeric Formulation of Extended-Release LAs

### Basic Knowledge on Polymeric DDS

Polymers are macromolecules which consist of thousands of repeated units (monomers). Unlike liposomes where Van der Waals’ force and hydrogen bonds play important roles, polymers are formed by covalent bonds, which provide better stability not only on shelf, but also when co-administered with free LAs. Large group of biocompatible and biodegradable polymers have been used in the fabrication of extended-release material, not only natural but also synthetic. Considering different chemical properties of amide and ester LAs, corresponsive loading methods can be used to optimize loading efficiency (electrostatic interaction, covalent conjugation, and encapsulation) [8]. Additionally, flexible morphologies such as nanoparticle, nanocapsule, nanogel, nanofilm, and nanofiber expand the application of polymeric DDS in practice when injection, dressing or film is needed [7, 8]. Furthermore, structural modifications empower polymeric DDS adjustable release profile such as days for perioperative pain, weeks for chronic pain, and co-delivery with a second drug to enhance LAs’ efficacy [7].

**Fig. 5:**
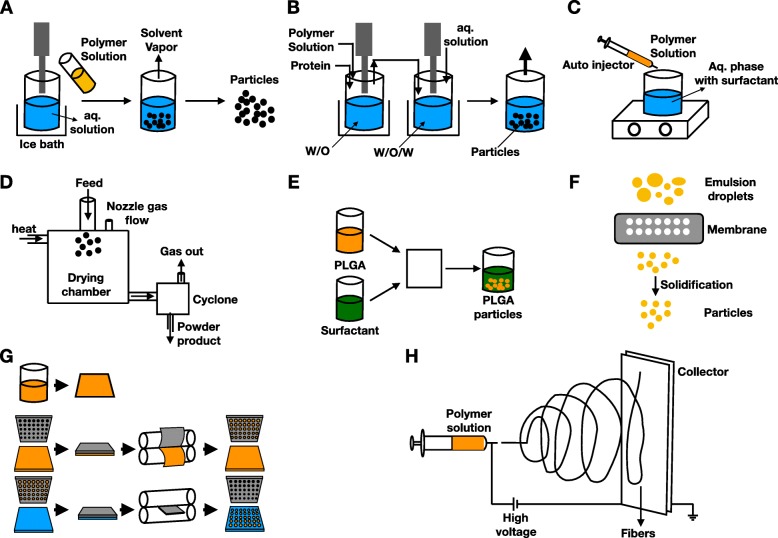
Classical techniques of polymersomes. **a** Single emulsion. **b** Double emulsion. **c** Nanoprecipitation. **d** Spray-drying. **e** Microfluidics. **f** Extrusion emulsification. **g** Particle replication in nonwetting template (PRINT). **h** Electrospinning

Among the most used synthetic polymers are polyesters. This category includes poly (l-lactide), poly (glycolic acid), poly (lactic-co-glycolic acid), and poly (e-caprolactone) [95]. Metabolites are usually small molecules such as carbon dioxide and water, which can be recycled or excreted safely [96]. On the other hand, production techniques have evolved greatly from traditional ones, such as double/single emulsion, precipitation, and spray-drying, into microfluidic platform, extrusion and particle replication in nonwetting template (PRINT) (Fig. 5a–g). Novel techniques such as electrospinning are also developed to enable flexibility in formulation and delivery (Fig. 5h) [95, 96].

There is another production method, self-assembly, which has not been utilized widely in drug delivery but may enlighten the future path of DDS. Self-assembly differs from other production procedures by spontaneous assembly into micelles of amphiphilic polymer-agent copolymers in aqueous environment. Therefore, it exhibits simplicity, high efficiency, and good water dispersibility [97, 98]. Copolymers can be formed through a variety of reactions, such as one-pot multicomponent reaction (mannish reaction between secondary amide and active hydrogen compound which are linked by formaldehyde) [97, 99], supramolecular reaction (host-guest interaction, such as cyclodextrin and adamantine) [100, 101], esterification and ring open reaction [102–104], thiol-yne click reaction [105], and post-modification of copolymers [106]. Although this technique is mainly used in fluorescent polymeric particle recently to help protect from fluorescence-quenching and achieve aggregation-induced emission, diverse reactions and interactions make it possible that different hydrophobic drugs can find responsive polymers (especially organic polymers) to self-assemble into micelles [107, 108]. For example, secondary amide in amide LAs can be used as active moiety reacting with polymers by mannish reaction. Alternatively, drugs can be absorbed into copolymers to achieve simultaneous imaging and treatment [101]. Since hydrophobic drugs are encapsulated in the core of micelles, it can provide a suitable inner environment for drug efficacy, such as alkaline environment for LAs. Meanwhile, great cell-uptake behavior may enhance drugs which target intracellular domains such as LAs [97, 100, 105]. Furthermore, reactions are usually catalyst-free/simple (such as microwave and ultrasound) [109, 110], solvent-economical, experimental-condition-mild, and time-saving, which empower its mass production in the future [97, 105].

Polymeric DDS releases active agents by several pathways. When water fluxes into polymers in the beginning, convection creates an osmotic force pumping drugs out. Yet, diffusion through pores formed in aqueous environment is the major way. It helps hydrophilic drugs, such as ionized LAs, to leak out. Contrarily, hydrophobic drugs can diffuse directly through. At the final stage of a typical tri-phase release profile, erosion of polymer dominates [111, 112].

Like liposomes, polymersomes also face the challenge of RES clearance. Morphology modification, stealth strategy, RES blockade, and macrophage depletion can be applied as well [40, 113–115]. Furthermore, the EPR effect facilitate tumor-target while auxiliary surface modification can help tissue specificity [43, 116]. Polymersomes do not induce significant systemic inflammation while some of natural polymers such as hyaluronan and laminin, and synthetic polymers such as negatively charged poly(lactic-co-glycolic acid) and polyurethane can reduce systemic inflammatory reaction [117–119]. Moreover, *N*-(2-hydroxypropyl) methacrylamide and polystyrene are reported to reduce neurotoxicity [120, 121].

### Diverse Morphology and Applications

Particle is a common form of polymeric DDS. Polymeric particles are generally divided into two categories: nanosphere and nanocapsule. Nanosphere is an evenly disperse system, while nanocapsule is an embedding drug in polymeric cavity [95]. The earliest researches built nanoparticles with poor entrapment efficiency (< 60%) and release duration (< 30 h) [122, 123]. With finesses of technology, the drug entrapment efficiency reaches nearly 90% [124–126], the highest result is 93.3% [127]. In-vitro release can reach a maximal duration of 35 days [128]. There is another available form termed nanofiber, which is produced by electrospinning. Electrospinning technique has been widely applied in drug delivery, but limited usage in LAs. There is one research only using electrospinning technique to fabricate bupivacaine-loaded suture. It gained satisfactory results on extended release (over 12 days), while significant analgesia appeared from day 1 to day 9 in rat skin wound model [129]. Mostly in polymersomes, drugs are entrapped physically; in polymer-drug conjugate, drug is bonded to polymer covalently to enhance drug loading capacity and prolong release furthermore [130]. Apart from solid forms, amorphous forms such as gel show more versatility and malleability. A 60-day release was seen in the work of Daryl Sivakumaran and his colleagues (Table 4) [134]. Rapid burst release over 60% of LAs was observed in the work of Haibo Qu and his colleagues (Table 4) [135].

**Table 4:** Summary of researches on traditional polymeric LAs delivery system

Reference Number	Polymer	LAs	Morphology	Diameter	Release profile	Analgesic efficacy
[122]	Poly (d, l-lactic acid)	Lidocaine	Nanosphere	250–820 nm	24–30 h	None
[123]	Poly (d, l-lactide-co-glycolide	Procaine hydrochloride	Nanoparticle	157.1–209.5 nm	> 6 h	None
[131]	Poly (d, l-lactide-co-glycolide	Ropivacaine	Nanosphere	162.7 ± 1.5 nm	None	None
[125]	Alginate/bis (2-ethylhexyl) sulfosuccinate and alginate/chitosan	Bupivacaine	Nanoparticle	926.5 ± 32 and 944.4 ± 98 nm	> 900min	Both of study groups prolong anesthetic efficacy compared to plain bupivacaine in paw withdrawal to pressure threshold in sciatic nerve blockade (*n* = 6–7, *p* < 0.01)
[124]	Poly (ε-caprolactone)	Benzocaine	Nanocapsule	149.4–209.2	None	None
[132]	Poly(l-lactide)	Benzocaine	Nanocapsule	120 nm	Up to 480 min	Prolonged the duration of analgesia in 0.006% or 0.06% benzocaine (BZC) up to 180 and 300 min respectively; increased intensity approximately twofold (*p* < 0.001), compared with 0.06% plain BZC in sciatic nerve blockade (*n* = 6–7)
[127]	Poly (ε-caprolactone)	Lidocaine	Nanosphere	449.6 nm	350 min	Nanospheric lidocaine significantly (*p* < 0.001) increased the intensity and duration of analgesia, compared with free lidocaine in sciatic nerve block (*n* = 5)
[133]	Alginate/chitosan	Bupivacaine	Nanoparticle	None	> 900 min	The total analgesic in infraorbital nerve blockade was improved by 1.4-fold (*p* < 0.001) with bupivacaine nanoparticle compared to bupivacaine(*n* = 7)
[133]	Poly(ε-caprolactone)	Lidocaine or prilocaine	Nanocapsule	None	> 18 h	Nanocapsule showed significant increase in duration of anesthesia (*p* < 0.01) and the maximum possible effect (*p* < 0.01), compared to the commercial formulation in tail flick test (*n* = 6)
[128]	Poly(lactide-co-glycolide)	Bupivacaine	Nanoparticle	150 ± 10 nm	35 days	Nanoparticle showed significant decrease on mechanically evoked response from day 1–14 days compared to blank particle
[134]	N-isopropylacylamide and acrylic acid	Bupivacaine	Gel	None	60 days	None
[135]	Tyrosine-polyethylene glycol-derived poly (ether carbonate) co-polymers and silica-based xerolgel	Bupivacaine or mepivacaine	Gel	None	7 days	Significant decrease of mechanical hypersensitivity in study group from 0.5 to 24 h compared to control and sham group (*p* < 0.05, *n* = 5)
[136]	Polysaccharide	Bupivacaine	Gel	None	120h	Significantly increased total force in study group (201.3) than LB group (144.4) to induce allodynia from 0–120 h (*p* = 0.0005, *n* = 20)
[129]	Poly (lactic-co-glycolic acid)	Bupivacaine	Nanofiber (suture)	800-1000nm	12 days	The greatest degree of analgesia was achieved at approximately 3 days, and significant relief was observed from 1 to 7–9 days compared to drug free suture group (*n* ≥ 6, *p* < 0.05=

In order to make the release intelligent, responsive materials are used in situations where structural changes, volume-phase transitions, or sol-gel transitions are needed [137]. Inflammatory reaction is a major and initial process when trauma happens. Along with it is the changes of pH and temperature. Thus, pH and temperature are stimuli used commonly in LAs delivery. In the work of Todd Hoare and his colleagues, thermal aggregation can help control regional accumulation of thermal-responsive nanogel. However, larger precipitates after aggregation induced more severe local inflammation (Table 5) [139]. Teresa Alejo and his colleagues used sequential heat pulse to trigger the collapse of nanoparticle, fastening profound release in a spatiotemporal way (Table 5) [138]. A pH-responsive polymer (methacrylicacid–ethyl acrylate) was used by Jeremy P.K. Tan to achieve decreased release of procaine chloride with decrease of pH (Table 5) [140].

**Table 5:** Summary of researches on responsive polymeric LAs delivery system

Reference number	Polymer	LAs	Diameter	Stimuli	Release profile	Toxicity
[138]	HGNPs@P(MEO2MA-co-OEGMA500)	Bupivacaine	206.5 ± 49.3 nm	Heat	After 12 sequential heat pulses, the cumulative diffusive release reached a 40% in less than 8 h compared to more than 160 h at 37 °C	At 2 mg/mL, the viability percentages obtained were between 31% (mMSCs) and 42% (U251MG)
[139]	Poly (*N*-isopropylacrylamide)	Bupivacaine	100–1000 nm	Heat	Thermally triggered aggregation happens from 32.4 to 37.5 °C	Nanogels had minimal impact on cell viability and large (> 500 nm diameter) nanogels typically remained as liquid-like residues in vivo and induced more severe inflammatory reactions
[140]	Methacrylicacid–ethyl acrylate (MAA-EA)	Procaine hydrochloride	30.9–141.1 nm	pH	The percentage of procaine released at pH of 8 was ∼ 90% compared to ∼ 30% at pH of 5	None

*HGNP* Hollow gold nanoparticle

Despite the advantages of being responsive to stimuli, pH and temperature-responsive polymers do not act precisely enough to localize therapeutic agents. Moreover, acidic environment and high temperature may bring harm. Therefore, stimuli which are able to modulate therapeutic agents precisely without harm are wanted. Although there is no application so far in LAs delivery, ultrasound-responsive nanoparticles exhibiting harmless property have shown precise localization in tumor targeting and imaging [141, 142]. However, ultrasound signal can be influenced by air attenuation and bone structure. Magnetic responsive nanoparticles exhibit broader application such as in respiratory system which is filled with air and central nervous system covered with cranial bone [143]. Furthermore, magnetic responsive nanoparticles can help spatially and temporally localize actives without restriction by tissues depth [143]. Generally, there are three mechanisms underlying magnetic response, which are magnetic deformation, magnetic guidance, and magnetic-induced hyperthermia [144, 145]. Aginate-based ferrogel undergoes pore formation with magnetic stimulation [146]. In contrast, chitosan-based nanoparticle undergoes pore formation with the help of heat produced by magnetic field [147, 148]. In order to decrease thermal damage to surrounding tissues, Wei Chen and his colleagues produced a core-shell structure to embed heat-producing core and prevent thermal damage [143]. The guidance function can help the nanoparticle migrate long distance and penetrate biological barrier [149]. Combination of aforementioned mechanisms can create synergistic outcomes [150].

Polymeric carriers endow LAs extended-release, but unexpected burst release does exist. Long-term degradation may induce foreign body response. Versatility of materials and mature techniques are advantages of polymeric carriers. Acidic metabolites, however, may hamper the effect of LAs. Although responsive polymers make delivery more intelligent, they do not solve the aforementioned problems above completely. Therefore, further improvements are needed.

### Lipid-Polymer Hybrid Carriers of Local Anesthetic

Liposomal and polymeric nanocarriers both have drawbacks such as low solubility, poor stability, undesired drug leakage. and diffusion [151–153]. Conventional liposomes are easily cleared out by enzymatic degradation and macrophage engulfing. Therefore, surface modification with inert and hydrophilic coats, such as PEG, ganglioside GM1, and phosphatidylinositol, have been used for protection against degradation by enzymes and macrophages. Furthermore, pegylation can enhance the negative charge of liposome and reinforce its attraction to cationic actives. In the work of Brett A. Howell and his colleagues, higher concentration and slower release of bupivacaine in human serum was observed in pegylated liposomes compared to conventional liposomes [154]. Alternatively, apolar cavity of biologically active compounds formed by cyclodextrins (a polymeric peptide), also known as an inclusion, in multi-vesicular liposomes can help achieve longer release. Inspired by various pioneer attempts to overcome drawbacks of liposome and polymeric nanoparticles, lipid-polymer hybrid nanoparticles come to the stage of controlled release [151, 155].

There are three systems of hybrid nanoparticles: lipid-core polymer-shell, polymer-core lipid-shell, and the polymer-lipid matrix. Lipid-core polymer-shell system using a polymeric coat improves the stability of liposome and delays clearance, while maintaining optimal biocompatibility of lipid core. The amphiphilic properties of some polymers can reduce surface tension of nanoparticles and decrease particle size to achieve better biocompatibility. Additionally, responsive polymers can enable liposomes more flexible behavior in various physical environment [152, 156]. Poly (acrylic acid) (Chol-PAA) is a pH-sensitive polymer, which tends to form globular structure from random coil in low pH. In poly (acrylic acid) (Chol-PAA)-caged liposome, the polymeric coat shrinks when environmental pH decreases, compressing core liposome to release significantly when liposome collapses [152]. Marina Sokolsky-Papkov and her colleagues used hybrid polymer (DL-lactic acid and castor oil) to formulate bupivacaine-loaded nanoparticles. This poly (fatty-ester) achieved relatively longer in-vitro release (beyond 1 week) and sensory blockade (> 72 h) compared to previous research [157]. Further research confirmed its release profile and extended its analgesic efficacy among thermal pain, mechanical pain, and rearing assessment [158]. Another research study explored the difference between oil nucleus on stability, release profile, and analgesia property, and found that a higher entrapment efficacy was observed compared to polymeric DDS [159].

Polymer-core lipid-shell system is commonly used in intravenous administration of drugs, especially anticancer drugs [151, 155]. The two components together help inhibit water infiltration and slow hydrolysis down, and as a result prevent drug diffusion and burst leakage. Lipid shell also enhances biocompatibility of nanoparticle with similarity to cell membrane [160]; this is promising in achieving simultaneous co-delivery of multiple drugs [151, 152, 160]. On the other hand, polymeric core can facilitate mechanical stability, shape control, and size distribution. Additionally, it can increase drug entrapment efficacy. In the work of Jianguo Wang and his colleagues, nanoparticle showed a slower release speed, smaller particle diameter, and higher drug loading compared to traditional liposomes [161]. The work by Pengju Ma at el. showed that hybrid bupivacaine nanoparticle had better stability, analgesic efficacy, and cytotoxicity [162].

The third system is a polymer-lipid matrix which can be subdivided into polymerized liposomes and nano-in-micro type. As their names suggest, polymerized liposome is a covalently bound liposome which demonstrates better stability and modulated release as mentioned above with basic knowledge on polymeric DDS [154], while the nano-in-micro system gathers nanoparticles into a matrix to achieve controlled release as shown in the work of Khanal Manakamana and his colleagues [163]. Moreover, hybrid nanoparticle was used in transdermal route showing better skin permeation than free bupivacaine in three studies by Yaocun Yue and Aimei Li at el., which expands its clinical application of analgesia [161, 164, 165].

## Future Perspectives

Nano-structured systems have been utilized in extended-release for drug delivery over decades. Tremendous breakthroughs have been seen in controlled release of LAs to achieve longer duration of analgesic and better safety profile, bringing in the successful approval of EXPAREL. At the same time, various modifications and combinations of nano-structured systems have broaden the horizon of LAs. With the guidance of physical or chemical stimuli, targeting LAs to treat the specific sources of pain may not be a difficult process anymore. Additionally, with the help of external and internal stimulus, we may modulate the release profile of LAs even after administration. Physiochemical properties of actives, rather than carriers alone, are also taken into account to formulate new and flexible systems. In the future, we may see precisely designed extended-release LAs to meet different demands of analgesic intensity, duration, and target sites in distinct surgeries, to make analgesia more individualized.

## Conclusions

LAs are key components in multimodal analgesia. Short duration and adverse side effects limit its application, which induces the emergence of extended-release LAs. Nano-structured DDSs show better biocompatibility and biodegradation compared to micro-structured DDSs due to similar size with physiological environment. Among various nanocarriers, liposomes achieve the first success in super-long-lasting LAs, which can release bupivacaine for 72 h in vivo. Liposomes also potentiate the safety of LAs with the protection of emulsion. The instability of liposome, however, hinders its storage and co-administration profile with additional free LAs. Compared to liposome, polymersome has a more advantageous profile with better stability and prolonged release. Moreover, the electrospinning technique and the stimuli-responsive property endow polymersomes with more flexibility in morphology and release behavior. Besides the optimization of materials and manufacturing processes, combination of nanocarriers is an alternative way to improve drawbacks and boost strengths. This is where hybrid nanocarriers come to the stage: hybrids not only improve the release profile but also broaden administration routes, such as the transdermal route. With the ever-emerging versatility of nanocarriers, extended-release may become more specific and controllable in the future to satisfy various analgesic demands.

## Data Availability

Not applicable.

## Abbreviations

BBB: Blood-brain barrier
CNS: Central nervous system
DDSs: Drug delivery systems
DPPA: 1,2-Dipalmitoyl-sn-glycero-3-phosphate
DSPG: 1,2-Distearoyl-sn-glycero-3-phospho-(1'-rac-glycerol)
EGFR: Epidermal growth factor receptor
EPR: Enhanced permeability and retention
FDA: American Food and Drug Administration
LAs: Local anesthetics
LAST: Local anesthesia systemic toxicity
PCA: Patient controlled analgesia
PEG: Polyethylene glycol
PRINT: Particle replication in nonwetting template
RES: Reticuloendothelial system
TAP: Transversus abdominis plane
VEGF: Vascular endothelial growth factor
